# Modeling Relapsing Malaria: Emerging Technologies to Study Parasite-Host Interactions in the Liver

**DOI:** 10.3389/fcimb.2020.606033

**Published:** 2021-01-29

**Authors:** Annemarie Voorberg-van der Wel, Clemens H. M. Kocken, Anne-Marie Zeeman

**Affiliations:** Department of Parasitology, Biomedical Primate Research Center, Rijswijk, Netherlands

**Keywords:** malaria, parasite-host interaction, plasmodium, cynomolgi, hypnozoite, relapse

## Abstract

Recent studies of liver stage malaria parasite-host interactions have provided exciting new insights on the cross-talk between parasite and its mammalian (predominantly rodent) host. We review the latest state of the art and and zoom in on new technologies that will provide the tools necessary to investigate host-parasite interactions of relapsing parasites. Interactions between hypnozoites and hepatocytes are particularly interesting because the parasite can remain in a quiescent state for prolonged periods of time and triggers for reactivation have not been irrefutably identified. If we learn more about the cross-talk between hypnozoite and host we may be able to identify factors that encourage waking up these dormant parasite reservoirs and help to achieve the total eradication of malaria.

## Introduction

Malaria, caused by *Plasmodium* parasites, remains a very serious infectious disease, killing over 400,000 people per year (WHO, 2019). With a complex life-cycle in mosquito and vertebrate hosts, the parasite has to interact with its hosts to be able to survive and multiply. In the vertebrate host, the parasite has life-cycle stages initially in the liver and subsequently in the blood. Five malaria species can infect humans, *Plasmodium 8falciparum, vivax, ovale, malariae* (Hay et al., 2004), and the recently added zoonotic parasite *P. knowlesi* (Yegneswaran et al., 2009). Most of the fatal malaria infections are caused by *P. falciparum*, but also infection with *P. vivax*, the second most important human malaria parasite, can result in death (Price et al., 2007). *P vivax*, as well as a few other primate malarias form dormant stages called hypnozoites in the liver, that after months or even years can re-activate to yield new malaria episodes, without new infections through mosquito bites. This hidden reservoir of parasites complicates future malaria eradication. Liver stage biology, and especially hypnozoite biology, remains obscure as the liver stages are relatively inaccessible. Here we review recent progress in studies on liver stage parasite-host interactions in general and zoom in on new technologies that will allow detailed biological studies on dormant liver stages and their interaction with the host.

## Parasite-Host Interactions Inside the Liver; the Rodent Models

The rodent malarias *Plasmodium berghei, Plasmodium yoelii, Plasmodium chabaudi*, and *Plasmodium vinckei* have played a pivotal role in understanding malaria biology [reviewed in (De Niz and Heussler, 2018)]. In the absence of prolonged blood stage cultures, these systems are almost exclusively *in vivo* based. Different mouse strains each have their own characteristics and the creation of transgenic mice has enabled studies that pinpoint specific parasite-host interactions (Liehl et al., 2014). The combination with highly efficient transfection systems for these parasite species (Mikolajczak et al., 2008; Matz and Kooij, 2015) renders the rodent malarias invaluable for studying parasite-host interactions *in vivo*.

Particularly liver stage research has greatly benefitted from the rodent models. Once sporozoites have switched from traversal to invasion (Coppi et al., 2007), the rodent malaria parasites show unprecedented multiplication inside hepatocytes. Within about 2 days, liver stage development is completed and thousands of merozoites are formed. Characterization of the liver stage parasites has been technically challenging due to their inaccessible location for experimentation. With robust *in vitro* parasite liver stage cultures established in many labs, more information has become available as to how the parasite manages to perform this daunting task.

The first comprehensive transcriptomic analysis for liver stages, which was combined with a proteomic survey, was described for *P. yoelii* (Tarun et al., 2008). The development of genetically engineered parasites allowed FACS isolation of liver stage infected hepatocytes. This revealed that liver stage schizonts express a wide range of metabolic pathways, including the liver stage-specific FASII pathway. Further in-depth RNAseq analyses performed at different timepoints during *P. berghei* liver stage development identified genes predominantly expressed in liver stages and showed that liver stage development was accompanied by differential expression of hundreds of parasite genes which may be regulated by a variety of posttranscriptional and posttranslational mechanisms (Caldelari et al., 2019; Shears et al., 2019).

While malaria liver stage development is a clinically silent phase of the life cycle, it has become clear that the hepatocyte does respond to the presence of the parasite in various ways. Transcriptional profiling of hepatoma cells early after infection with *P. berghei* showed an initial stress response of the cell to the presence of the parasite, which was followed by altered host cell metabolic responses to meet the requirements of parasite multiplication while maintaining parasite survival (Albuquerque et al., 2009). The parasite appears to prolong survival of the host cell by protecting it against extrinsic apoptosis (van de Sand et al., 2005; Kaushansky et al., 2013a), for example by suppression of host cell p53 (Kaushansky et al., 2013b) and by upregulating the “cellular inhibitor of apoptosis” protein (cIAP) (Ebert et al., 2020). Furthermore, the parasite has developed mechanisms to protect itself against elimination by autophagy of the host cell (Prado et al., 2015; Agop-Nersesian et al., 2017; Real et al., 2018). While autophagy can have detrimental effects, the liver stage parasite also appears to benefit from a non-canonical form of autophagy, termed *Plasmodium* Associated Autophagic-Response (PAAR) which was shown to support liver stage development (Agop-Nersesian et al., 2017; Coppens, 2017; Wacker et al., 2017; Evans et al., 2018).

That the malaria parasite is sensed by its host has also become evident by the specific type I interferon response that is triggered by liver stage malaria parasites following rodent liver infection (Liehl et al., 2014). In a rodent malaria model, it was shown that this response could inhibit malaria reinfections (Liehl et al., 2015). Similarly, host responses elicited during blood stage infection may impair liver stage infection. This appears to be mediated by the iron-regulatory hormone hepcidin, which restricts iron availability in the liver and thereby inhibits liver stage growth of the parasite (Portugal et al., 2011). This points to the importance of metal homeostasis during liver stage development, which is also highlighted by detrimental effects on liver stage parasites caused by gene knockouts of parasite metal transporters (Sahu et al., 2014; Kenthirapalan et al., 2016).

The parasite has been shown to recruit various host cell proteins in order to sustain its development, including GLUT1 (Meireles et al., 2017), aquaporin-3 (Posfai et al., 2018; Posfai et al., 2020) and protein traffic modulators such as COPB2 and GGA1 (Raphemot et al., 2019). Many of the host factors involved are recruited to the host-parasite interface in the liver cell, the parasitophorous vacuole membrane (PVM). Most interactions between host cell proteins and parasite proteins located at the PVM have remained elusive. To date, only a few connections between parasite antigens and hepatocyte proteins have been described. “Up-regulated in sporozoites protein” UIS3 has been shown to interact with liver fatty acid binding protein 1 (LFABP1) (Mueller et al., 2005; Mikolajczak et al., 2007), suggesting fatty acid scavenging from the host cell. Furthermore, *P. berghei* Exported protein 1 (EXP-1) was shown to interact with host Apolipoprotein H (ApoH) (Sa et al., 2017). These interactions have only been described for rodent malaria and possibly different parasite-host combinations require different interactions. This is highlighted by the finding that *P. falciparum* EXP-1 did not appear to interact with human ApoH (Sa et al., 2017).

Clearly, rodent models have been and will continue to play an important role in dissecting various aspects of malaria liver stage biology. However, it will be important to determine how the findings in these models translate to the human parasites, as has already been done for only a limited set of proteins, such as aquaporin-3 (Posfai et al., 2020) and Mucin-13 (LaMonte et al., 2019). While the rodent malaria studies allow investigations into the biology of developing liver stages, these parasite species do not develop into hypnozoites and thus do not enable studies on hypnozoite biology and hypnozoite-hepatocyte interaction.

## *P. vivax* Parasite-Host Interactions Inside the Liver

Studying *P. vivax* parasite-host interactions in the liver is hampered by the fact that *P. vivax* develops only in primates. *P. vivax* liver stages were studied in humans, as described by Shortt and Garnham in 1948 (Shortt et al., 1948), in which liver biopsies were taken from a volunteer at day 6/7 post infection by the bites of ~1700 mosquitoes. They demonstrated the existence of a liver tissue stage in the *P. vivax* malaria life-cycle, similar to their observations in monkeys infected with *P. cynomolgi* sporozoites that they published shortly prior to the human experiment (Shortt and Garnham, 1948a). They observed large liver schizonts responsible for primary disease, but unfortunately did not find the hypnozoites, which were described only in 1980 by Krotoski (in liver biopsies from *P. cynomolgi*-infected monkeys) (Krotoski et al., 1980). A lot of knowledge has been gained from early experimental human infections regarding relapse patterns and the clinical profile of *P. vivax*, but these types of experiments are nowadays restricted. Although sporozoite-derived controlled human infections with *P. vivax* are allowed (under strict supervision) and can be highly significant to test new drugs or vaccines, volunteers are usually cured at low blood stage parasitemia (Herrera et al., 2011; Arevalo-Herrera et al., 2016). Relapses are not studied in this model and studying the liver stage parasites by taking liver biopsies from these volunteers is not performed. The best non-human model for *P. vivax* infections used to be the chimpanzee, and after finding the first hypnozoites in *P. cynomolgi*-infected rhesus monkeys, *P. vivax* hypnozoites were discovered in liver biopsies taken from *P. vivax*-infected chimps (Krotoski et al., 1982a). Nowadays animal experiments on apes are banned, so this model is no longer available (Hutson, 2010).

Other primate models for *P. vivax*, like Saimiri or Aotus monkeys have been primarily used for schizonticidal drugs and vaccine efficacy studies [reviewed in (Joyner et al., 2015)] but to be able to detect (low level) blood stage parasites caused by relapses animals need to be splenectomized, and pattern and frequency of relapses appear to be difficult to predict in these animal models (Joyner et al., 2015).

An important step for *in vivo P. vivax* research was achieved by the development of the FRG huHep chimeric mouse. This model is suitable to study the pre-erythrocytic stages of *P. vivax*, including hypnozoites, and can be used to test the activity of potential radical cure drugs that would kill hypnozoites (Mikolajczak et al., 2015). Blood stage parasites can be observed when injecting the FRG huHep mice with human reticulocytes around the time that the merosomes will be released from the mature liver schizonts, but this becomes very complicated if one wants to study relapses. Also, these mice are severely immunocompromised and will not completely reflect the human response to malaria infection or drug treatment. Thus, this model may not be suitable to study parasite-host interactions of relapsing malaria.

Studying parasite-host interactions *in vitro* may sound a bit counterintuitive, but some processes can be fairly easily studied in an *in vitro* setting, like the hepatocyte’s response to infection, activity of anti-relapse compounds and monitoring reactivation of the dormant stages. Primary human hepatocytes or hepatoma-derived cell lines (Hollingdale et al., 1984; Mazier et al., 1984; Sattabongkot et al., 2006; Chattopadhyay et al., 2010) are used as monoculture to study *P. vivax* liver stages *in vitro*. Recently, Roth et al. (Roth et al., 2018) have described a system using cryopreserved human primary hepatocytes and patient-derived sporozoites with high infection rates. This system should be helpful in the identification of new hypnozoite targeting compounds, as well as studying hypnozoites and reactivation as well as hepatocyte responses to infection. However, all of these models are dependent on patient material for the infection of mosquitoes. This means that there can be large variation between the different experiments, both in infection rate as well as in hypnozoite ratios (Roth et al., 2018). The variation caused by the different lots of hepatocytes is reduced when using cryopreserved cells, but variation caused by different patient-derived parasite isolates can’t be tackled. This can be circumvented by using the *P. cynomolgi*-monkey model for relapsing malaria (described below). Additional advantages for this model are that *in vitro* and *in vivo* experiments can be performed with the same well-characterized parasite and the availability of a robust transfection procedure for this parasite. *P. cynomolgi* can be genetically modified using episomes, centromere-containing constructs (for stable retention of the episome) and by single crossover integration into the genome (Kocken et al., 1999; Akinyi et al., 2012; Voorberg-van der Wel et al., 2013; Voorberg-van der Wel et al., 2020). Transfection of *P.vivax* is also possible, but is more difficult due to the restrictions (as described above) when working with small monkeys like Aotus and Saimiri (Pfahler et al., 2006; Moraes Barros et al., 2015), and so far the papers describing *P. vivax* transfection only show proof of concept.

## *P. cynomolgi*, the Monkey Sister Parasite of *P. vivax*

The monkey malaria parasite *P. cynomolgi* is considered to be an important model for the relapsing human malaria *P. vivax*, as it is phylogenetically closely related (Tachibana et al., 2012) and shares many biological characteristics (**Table 1**). Not only liver stage parasites, but also hypnozoites were first identified in the liver of rhesus monkeys that had been infected with high numbers of *P. cynomolgi* sporozoites (Shortt and Garnham, 1948a; Krotoski, 1985).

**Table 1:** Comparison of biological characteristics of *P. vivax* and *P. cynomolgi*.

	*P. vivax*	*P. cynomolgi*
**Characteristics life cycle**		
Asexual blood stage cycle	48 h (Garnham, 1966)	48 h (Garnham, 1966)
	Schüffners dots (Garnham, 1966)	Schüffners dots (Garnham, 1966)
Early development gametocytes (time to maturation)	Yes, 2–3 days (Bousema and Drakeley, 2011; Ngotho et al., 2019)	Yes, 58 h (Hawking et al., 1968)
Invasion	Reticulocytes (Noulin et al., 2013)	Reticulocytes (in humans; in monkeys also normocytes) (Kosaisavee et al., 2017)
Pre-erythrocytic stage	8 days (Fairley, 1946)	8–10 days (Shortt and Garnham, 1948b)
Hypnozoites	Yes (Krotoski et al., 1982a)	Yes (Krotoski et al., 1982b)
Relapse pattern	Short latency and long latency (White, 2011)	Short latency (Schmidt, 1986)
**Research tools**		
In vitro blood stage culture	No	Yes (but no transmission yet) (Chua et al., 2019b)
In vitro liver stage culture	Yes (Gural et al., 2018b; Roth et al., 2018)	Yes (Dembele et al., 2011)
In vivo drug screening model	Limited (Mikolajczak et al., 2015)	Yes (Schmidt, 1983)
In vivo relapse model	Anecdotal (Joyner et al., 2015)	Yes (Schmidt, 1986)
Transfection technology	Proof of concept (Pfahler et al., 2006; Moraes Barros et al., 2015)	Episomes; centromeres; single crossover (Kocken et al., 1999; Akinyi et al., 2012; Voorberg-van der Wel et al., 2013; Voorberg-van der Wel et al., 2020)
Genome sequenced	Yes (Carlton et al., 2008)	Yes (Tachibana et al., 2012; Pasini et al., 2017)

Monkeys infected with *P. cynomolgi* sporozoites have shown similar pathology as *P. vivax-*infected humans, including anemia and thrombocytopenia (Joyner et al., 2016; Joyner et al., 2017). In addition, it was shown that *P. cynomolgi* relapses can be clinically silent. This is likely to be due to the rapid development of memory B cell responses that help to clear asexual blood stage parasites but not gametocytes (Joyner et al., 2019).

Moreover, *P. cynomolgi* showed drug activity profiles that were highly similar to *P. vivax* (Schmidt et al., 1982b). This led to large scale drug screening studies with *P. cynomolgi* sporozoite-induced infections in rhesus monkeys as central step in efforts [which initially also used patients undergoing *P. vivax* malaria therapy as well as prison inmate volunteers (Coatney, 1985)] to find new hypnozoite-killing drugs (Davidson et al., 1981; Schmidt et al., 1982b; Schmidt, 1983; Dutta et al., 1989; Deye et al., 2012).

The *in vivo* data have been crucial for the discovery of the liver stages and for assessing the effects of drugs targeting these stages. However, experimentation and throughput are limited for ethical and economic reasons and, apart from the 8-aminoquinolines, other compounds killing hypnozoites have not been identified. Therefore, higher throughput approaches in order to find new, more potent and less toxic drugs that cure relapsing infections are needed (Wells et al., 2010; Campo et al., 2015). Knowledge of liver stage biology may reveal new targets for drug development, which may be more efficient than random screening approaches.

The advent of *in vitro* culture techniques for malaria liver stage parasites, including *P. cynomolgi*, (Millet et al., 1988) has greatly increased opportunities for the development of drug screening platforms and to begin to study parasite-host interactions. For *P. cynomolgi*, a low-throughput 96-well based assay system which enabled testing of compounds that are active against hypnozoites was developed, in which hypnozoites could be distinguished from developing forms (schizonts) by their size and differential sensitivity against selected drugs (Dembele et al., 2011).

Using this assay, a PI4 kinase inhibitor (McNamara et al., 2013) was identified showing high activity against early hypnozoites (Zeeman et al., 2014). This translated to *in vivo* prophylactic, but not radical cure activity (Zeeman et al., 2016), illustrating that young hypnozoites may be different from maturing hypnozoites. This is in line with earlier *in vivo* work which showed that when *P. cynomolgi* infected rhesus monkeys were treated at different timepoints with only 1 or 2 dosages of primaquine, it appeared that some phases (mainly early stages) of liver stage development were more vulnerable for the activity of the drug than others (Schmidt et al., 1982a).

The culture system was further improved through the addition of a Matrigel cover, which makes it possible to culture the *P. cynomolgi* exoerythrocytic forms for prolonged periods of time, revealing possible events of hypnozoite reactivation (Dembele et al., 2014). Recently, a 3D spheroid-culture system was reported that allows long-term cultivation of *P. cynomolgi* liver stages including full maturation of liver schizonts and invasion of red blood cells. While mimicking the *in vivo* microenvironment of the liver the 3D-structure of the spheroids renders it difficult to image and quantitate parasite load, presenting an obstacle for the use of this technology for high-throughput screening (Chua et al., 2019a). However, such a 3D-platform may be suitable for studying parasite-host interactions, with optimal *in vitro* hepatocyte quality, mimicking the *in vivo* situation.

In an attempt to characterize hypnozoites at the transcript level, *P. cynomolgi* day 7 hypnozoites and schizonts were collected by Laser Capture Microdissection (LCM) (Cubi et al., 2017). Two hypnozoite samples were obtained, containing a total of 45 and 59 hypnozoites, respectively (Cubi et al., 2017). Given the low levels of hypnozoite RNA in these small-sized samples, low read counts were obtained. Some ApiAP2 transcription factors were identified that were upregulated in hypnozoites. Further functional studies are needed to confirm the roles of these proteins.

*P. cynomolgi* has the advantage that it can be genetically manipulated (Kocken et al., 1999; Akinyi et al., 2012). By including a centromere (Iwanaga et al., 2010) in the construct, reporter lines have been developed which enable live visualization and purification of hypnozoites and liver stage schizonts (Voorberg-van der Wel et al., 2013). This has allowed a comprehensive transcriptomics analysis of day 6/7 and day 9 hypnozoites and schizonts (Voorberg-van der Wel et al., 2017; Bertschi et al., 2018). This revealed that developing schizonts are metabolically highly active, while hypnozoites continue to shut down transcription, except for pathways involved in the maintenance of genome stability, glycolysis and the pentose phosphate pathway. A marker for hypnozoites was not identified but Liver Stage Protein-2 was found to be schizont-specific and to be expressed very early on during schizogeny (Gupta et al., 2019). Rhesus host responses to *P. cynomolgi* infection and development in cultured hepatocytes have not yet been reported.

Using a *P. cynomolgi* reporter line that constitutively expresses GFP and shows mCherry expression when schizogony occurs, reactivation of hypnozoites *in vitro* was observed (Voorberg-van der Wel et al, 2020). This provides strong proof for the hypnozoite theory of relapse and allows screening of compounds that induce activation. If such compounds can be identified, “wake-and-kill” strategies can be envisaged in which hypnozoite activation is evoked, followed by killing of developing forms by currently available drugs.

The trigger for hypnozoite activation has remained enigmatic (**Box 1**) and the parasite-host interactions involved are elusive. Hypnozoite activation may be epigenetically controlled (Dembele et al., 2014). Furthermore, it has been suggested that activation may be triggered by mosquito bites (Hulden and Hulden, 2011), infectious disease (Shanks and White, 2013; Commons et al., 2019), or blood transfusion (Shanks and Waller, 2019). The *P. cynomolgi* fluorescent reporter line now offers the opportunity to investigate if/which molecules may stimulate hypnozoite activation. It must be realized, however, that in the context of the current *in vitro* platform it may be difficult to mimic the complex bodily reactions possibly involved in this. Moereover, reactivation events in culture are rare, making it challenging to isolate reactivating hypnozoites to study parasite and host transcriptomics.

Another question mark is how the hypnozoite survives for such a long time in a hepatocyte. Under normal conditions, the life-span of hepatocytes is estimated to be 6–12 months (Seeger and Mason, 2000). If the late recurrences [800–1,000 days after infection; (Schmidt, 1986)] of *P. cynomolgi* sporozoite induced infections in rhesus monkeys derive from activated hypnozoites, then how is this possible? Does the hypnozoite extend the longevity of the hepatocyte, or does it end up in a new hepatocyte after cell division?

Box 1 **Outstanding questions:**

Hypnozoite-host interaction

*How do the findings in the rodent liver stage models with respect to parasite-host interactions relate to the primate malarias?

*Which parasite-host interactions occur in the liver stage development of primate malarias?

*What are the differences between *in vitro* and *in vivo* parasites in terms of parasite-host interactions?

*How does the hypnozoite hide from the host immune system?

Hypnozoite-dormancy

*Do hypnozoites preferentially develop in a certain type of hepatocyte?

*How can a hypnozoite remain in the liver for prolonged times (longer than the generally estimated lifespan of hepatocytes)

*When does hypnozoite commitment occur?

*Which parasite/host molecules are involved in maintaining hypnozoite dormancy?

Hypnozoite-reactivation

*What is the mechanism behind hypnozoite activation?

*Is there a trigger involved or is it stochastic, *via* a biological clock, or a combination of this?

## Newly Emerging Technologies

**Figure 1:**
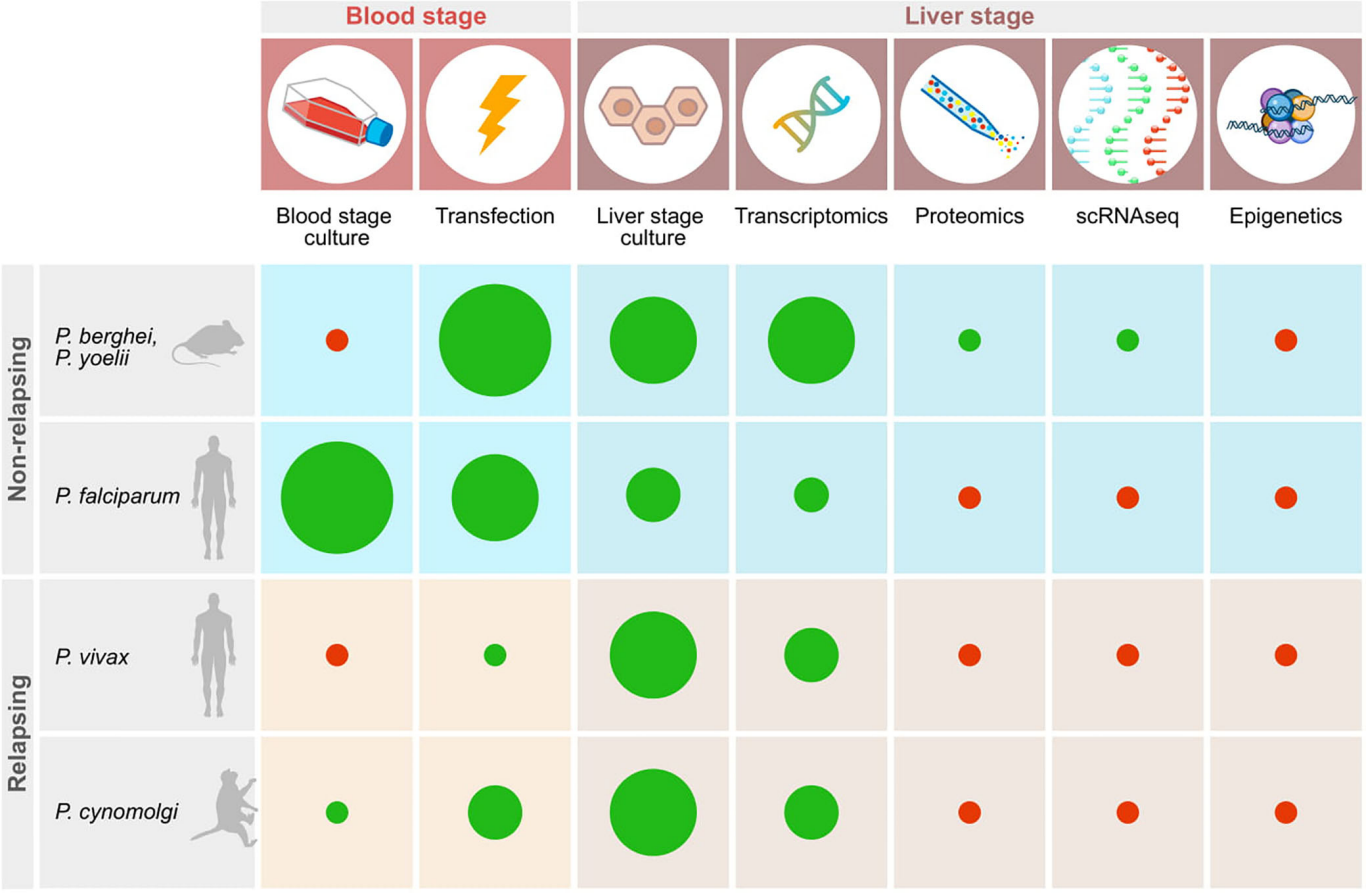
Schematic representation of important technologies available for studying relapsing and non-relapsing malaria parasites. Red dots indicate the absence of tools, green dots show that techniques have been established. The size of the diameter of the dots schematically indicates how widely the technology has been adopted based on published reports.

Liver stage parasites reside inside hepatocytes, located inside the liver. Given this multilayered, inaccessible location it has proven difficult to study this stage of the parasite life cycle. Furthermore, the existence of two forms of the parasite in some primate species, hypnozoites and schizonts, adds another layer of complexity to this. Much knowledge of parasite-host interactions of liver stage parasites has already been gained in the rodent malarias, although most likely this information represents only the tip of the iceberg. It will be important to determine whether this information can be translated to the primate malaria species. On top of this, virtually nothing is known about the interactions that take place between the hypnozoite stage of development and its host cell (**Figure 1**). Tools to study this are vital and have only recently begun to emerge, benefitting from technologies that have already been developed for the rodent malarias.

The development of liver stage cultures has greatly facilitated liver stage research. Some systems use hepatoma-derived cell lines (Hollingdale et al., 1984; Mazier et al., 1984; Sattabongkot et al., 2006; Chattopadhyay et al., 2010). While this provides a constant source of host cells, these cells differ in a number of aspects from primary hepatocytes (Tripathi et al., 2020), including a lower metabolic activity (Castell et al., 2006) and a high dependence on glucose uptake (Meireles et al., 2017). Therefore, care should be taken to validate results that mimic the natural situation. The importance of metabolic activity of hepatocytes was investigated for *P. falciparum* (Yang et al., 2020 BioRXiv, non peer-reviewed paper). This study indicates that *P. falciparum* liver stage development is strongly influenced by the differential metabolic activity of human hepatocytes derived from different zones of the liver.

The drawback of cultures using primary hepatocytes is that after about 12 days of culture the hepatocyte quality starts to deteriorate (Voorberg-van der Wel et al., 2020), which precludes analyses of hypnozoite activation. Approaches to overcome this issue include the addition of a Matrigel cover (Dembele et al., 2014), co-cultivation of human primary hepatocytes with fibroblasts (March et al., 2013; Gural et al., 2018a) or through the use of specific 384-well plates coated with collagen (Roth et al., 2018). Nucleic-acid mediated gene silencing has been successful in this type of systems, having the potential of exploring functional parasite-host interactions (Mancio-Silva L et al., 2019).

The advent of three-dimensional (3D) cell culture methods has opened up ways to develop cultures that mimic the *in vivo* physiological conditions to a greater extent. Proof-of-concept of this type of technology has already been shown, involving the use of hepatic spheroids using various hepatoma cell lines for culturing *P. berghei* (Arez et al., 2019) and using primary hepatocytes for *P. cynomolgi* (Chua et al., 2019a). Full development was shown for both parasite species, and cultures could be maintained for prolonged periods of time [up to 60 days in case of the simian spheroids (Chua et al., 2019a)]. While further improvements in terms of infection rate and by adding more cell types to create organoid like features is warranted, this type of systems provide new opportunities to study hypnozoite activation *in vitro* under conditions that are resembling *the vivo* situation.

More continuous, stable sources with truly hepatocyte features may be derived using newly emerging stem cell technologies. Proof of concept liver stage infections have already been shown using human induced Pluripotent Stem Cell (iPSC) derived hepatocyte-like cells (Ng et al., 2015) and chemically differentiated mouse embryonic stem cell (ESC)-based cells (Tripathi et al., 2020). These systems are attractive, since they not only provide a virtually unlimited source of hepatocytes, but the stem cells are also amenable to genetic manipulation thus allowing validation of genes important for parasite-host interactions in liver cells. In this way it was shown that the host adipose triglyceride lipase gene was dispensable for *P. berghei* liver stage development (Tripathi et al., 2020).

Little is known about host molecules involved in hypnozoite/liver stage development. Given that the first hypnozoite transcriptomes have become available (Cubi et al., 2017; Voorberg-van der Wel et al., 2017; Bertschi et al., 2018), a Dual-RNAseq approach can be envisaged whereby not only the transcriptome of the parasite, but also that of the host cell can be determined (LaMonte et al., 2019).

Information about transcriptional profiles of individual parasites can be obtained by a new technique called single cell RNA sequencing (scRNA-seq). Although technically challenging, researchers have accomplished (Poran et al., 2017) and optimized (Reid et al., 2018) a method for single cell RNA sequencing of malaria parasites. Using this new technique, individual parasites of all stages of the *P. berghei* life cycle were sorted and a transcriptional profile was generated, including difficult samples such as rings, which have low levels of RNA, and ookinetes, which are hard to sort (Howick et al., 2019). When application of scRNA-seq and other newly emerging “omics” approaches [e.g. lipidomics, metabolomics, proteomics, epigenomics (Cowell and Winzeler, 2018)] to relapsing malaria species (and more specifically to dormant liver stages) becomes feasible, these studies will likely shed more light onto genes involved in hypnozoite dormancy/activation.

The capacity to genetically modify parasite genes is key to study genes that may be involved in parasite host-interactions essential for hypnozoites. At the moment, such studies can only realistically be envisaged using the primate malaria *P. cynomolgi*. Lines that express reporter genes in *P. cynomolgi* liver stages have already been engineered (Voorberg-van der Wel et al., 2020), opening up studies that investigate phenotypic consequences of overexpression of gene candidates that may be involved in hypnozoite development. However, the *P. cynomolgi* transfection system is still in its infancy and only limited studies have been reported. Further development is warranted, because full exploitation of the capacity to genetically modify a relapsing parasite species will be vital for studying parasite-host interactions of hypnozoites with their host cell. Transfection systems have already been further optimized in other malaria species and it is expected that tools successful in these parasites, such as Crispr/Cas9 gene modification reviewed in (Lee et al., 2019), conditional (over)expression using DiCre (Jones et al., 2016) will be applicable to *P. cynomolgi* as well. Development of transfection tools may greatly benefit from the recently developed blood stage culture for this parasite (Chua et al., 2019b), extending the range of conditions that can be tested avoiding the use of donor and recipient monkeys.

## Author Contributions

All authors listed have made a substantial, direct, and intellectual contribution to the work and approved it for publication.

## Funding

Some of the work presented here was supported by funding from the Medicines for Malaria Venture, and the Bill and Melinda Gates Foundation, grant number OPP1141292.

## Conflict of Interest

The authors declare that the research was conducted in the absence of any commercial or financial relationships that could be construed as a potential conflict of interest.

## Abbreviations

P., plasmodium; FACS, fluorescence-activated cell sorting; FASII, fatty acid synthesis (in apicoplast); RNAseq, Ribonucleic acid sequencing; PVM, parasitophorous vacuolar membrane; UIS3, Up-regulated in sporozoites; huHep, human hepatocytes; 3D, three dimensional.
